# The impact of the single contract on health service delivery in the Democratic Republic of the Congo (DRC): findings from a quasi-experimental study

**DOI:** 10.1038/s44401-025-00053-0

**Published:** 2025-12-16

**Authors:** Wu Zeng, Marion Cros, Michel Muvudi, Linda Mobula, Dominique Baabo, Hadia Samaha, Fatima El Kadiri El Yamani

**Affiliations:** 1 Georgetown University, Washington, DC USA; 2 Heller School for Social Policy & Management, Brandeis University, Waltham, MA USA; 3 World Bank Country Office in India, New Delhi, India; 4 World Bank Country Office in the Democratic Republic of the Congo, Kinshasa, Democratic Republic of the Congo; 5 World Bank, Washington, DC USA; 6 Project Implementation Unit of World Bank Health Project, Ministry of Public Health, Hygiene and Prevention, Kinshasa, Democratic Republic of the Congo

**Keywords:** Health policy, Health services

## Abstract

The Democratic Republic of the Congo (DRC) initiated a contract (single contract) among the Provincial Ministry of Public Health, Provincial Health Divisions (PHDs), and development partners to strengthen the function of PHDs. Using a quasi-experimental research design, we assessed the impact of the single contract on primary health care (PHC) delivery in the country. We found that the single contract had a no direct impact on PHC services. When combined with the performance-based financing (PBF) program, the single contract demonstrated a more favorable impact on delivering PHC services. The single contract, though promising from a resource coordination and governance perspective, necessitates supplemental policies/interventions, such as PBF, to build an amiable health ecosystem to facilitate its effect on service delivery.

## Introduction

Efforts and commitments to achieving universal health coverage (UHC) in the Democratic Republic of the Congo (DRC) face significant challenges, despite the considerable progress made. The challenges to achieving UHC in DRC are multiple and include, but are not limited to, the following: inadequate health coverage for primary health services, low per capita health expenditure, a high financing gap for health, a shortage of qualified human resources for health, and high out-of-pocket expenditure for health, which lead to poor population health status in the country (e.g., high maternal and infant mortality rates)^1^. The coverage of essential health services remains inadequate. In 2009, although 79% of health zones had a health facility offering a minimum package of primary health services, only 12% of health facilities offering childbirth services provided basic emergency obstetric services, and only 6% of health centers offered a complete basic package of primary health care services that met standards^2^. Overall, the delivery of primary health care in DRC was weak.

To improve the governance of health services, DRC has implemented a series of decentralization reforms since 2006^3^. Under the decentralization, the Ministry of Public Health, Hygiene, and Prevention (MoPH) plays a normative role and defines the health policies, strategies, standards, and guidelines, while the provincial health system (PHS) in DRC plays an important role in governing and delivering primary health services in health zones. The PHS led by the provincial Ministry of Health comprises the Provincial Health Division (PHD), the Provincial Health Inspectorate, the Regional Distribution Center for Drugs, the provincial hospital, and other provincial health structures^4^. The PHS organizes, coaches, and manages the primary health care provided by health zones—corresponding to districts in many other African countries. PHDs are tasked to provide technical supervision and translate directives, strategies, and policies in the form of instructions to facilitate the implementation of actions in the health zones. Currently, DRC has 26 PHDs, corresponding to the 26 jurisdictional provinces. With the decentralization, the earmarked budget allocation for health transferring to provinces also increased substantially. Yet, the execution of transfers remains a concern, due to reasons such as incomplete decentralization and limited provincial budget^5^.

Additionally, PHDs are responsible for coordinating donor funding within the province. DRC’s health system heavily relies on donor funding, with substantial financial support from donors^6^. The fragmentation of financing from financial partners supporting health zone activities had hindered the achievement of the National Health Development Plan results^2^. It was reported that PHDs were inefficient in aligning development partners’ prioritizations to the nation’s^7^, primarily due to the low capacity of human resources and shortage of financial resources at PHDs^8^. PHDs were blamed for not being able to make optimal use of the different sources of financing, given the multitude of contracts (between provincial health directorates and development partners, or zonal health authorities and development partners) and the different development partner financial procedures involved^9^.

To address the concerns of the fragmentation and capacity of PHDs, local and international actors organized brainstorming workshops– particularly the Matadi workshop in 2015– which helped design and implement the “Single Contract”^4^. The single contract, developed in 2017, is the contract among the Provincial Ministry of Public Health (PMoPH), the PHD, and development partners. Under the single contract, development partners (e.g., World Bank, Gavi, and Global Fund) are mandated to provide financial support for the administrative costs of PHDs according to the negotiated budget that was linked to services. As obligations, PHDs are mandated and held accountable for implementing various management activities related to strengthening health service deliveries in the province. The main management activities include coordination, supervision, and provision of technical support to health zones related to health service delivery^4,10^. To monitor the implementation of the single contract, the PMoPH provides a quarterly evaluation of the performance of the contract and shares evaluation results to enhance transparency. The single contract also includes “a single performance framework with indicators focused on the health sector management and coordination at the provincial level, cross-sectoral collaboration, technical support and coaching at the health zone level, as well as resource management and health information management”^4^.

**Fig. 1:**
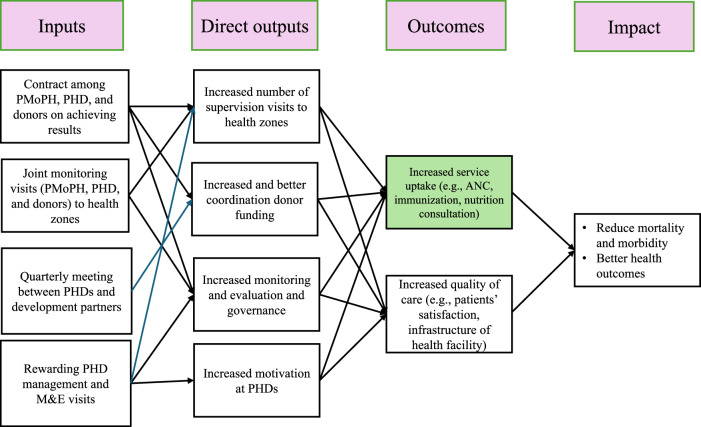
Logical framework for the single contract. ANC Antenatal Care, M&E Monitoring and Evaluation, PHD Provincial Health Division, PMoPH Provincial Ministry of Public Health.

The key elements of the single contract aim to strengthen the provincial health system by providing financial support to the PHD and linking the financial support to health system performance indicators through the contract to enhance the PHD’s core functions. These elements are essentially related to health service delivery. Thus, we hypothesized that the single contract would affect health service delivery through enhanced funding coordination among development partners, strengthened planning, and frequent supervision and monitoring if the single contract program continues to function and achieve its intended objective of tightening the PHDs’ function. Fig. 1 shows the logical framework for the single contract’s impact on health service delivery. The logical framework starts with interventions applied to different stakeholders under the single contract (e.g., contract among PMoPH, PHD, and donors, as well as joint monitoring visits). For each of the interventions, the framework specified its potential effects on direct outputs (e.g., number of joint visits), outcomes (e.g., improved uptake of health services and quality of care, and long-term impact (e.g., reduced mortality and morbidity). It should be noted that the single contract focuses on enhancing PHDs’ accountability and transparency via contractual agreements and performance-based financing (PBF). Numerous factors, however, may influence the conversion of these process measures into tangible outputs, outcomes, and impact.

The single contract has been implemented for more than six years, and we found only two evaluation studies in DRC. These two studies used a qualitative approach to assess the impact of the single contract on process measures^10,11^, showing the positive effect of the single contract on PHD’s management activities. For example, Bisimwa et al. found that the single contract contributed to improved planning and management in using health resources, strengthening the technical and financial partners between the PHD and technical financial partners (TFPs)^10^. Mopene et al. found a strong support from TFPS on the single contract and that the single contract helped the better alignment of partners for funding PHD’s activities although the authors claimed that the weak financing by the government remained a concern^11^.

Despite favorable qualitative findings of the single contract on its implementation process, there is no study quantitatively examining the potential impact of the single contract on PHC service delivery in DRC. To fill this gap, this study used a quasi-experimental design to assess the single contract’s effect on delivering PHC services in the country.

## Results

### Descriptive analysis of output indicators

The left side of Table 1 shows the log of the average services and the mortality at hospitals per quarter as well as the quarterly growth rate (QGR) of indicators in the provinces without the single contract. It reveals that all health services were increased from 2017 to 2021. The service with the largest increase included nutrition consultations, with a QGR of 6.7%. The lowest increase was in curative visits, estimated at a 1.7% increase. The mortality rate decreased steadily, with a 0.7% reduction per quarter. The combined service index had a growth rate of 2.9% per quarter. All service growth rates, as well as the quarterly reduction rate of mortality at hospitals, were statistically significant (P < 0.05).

**Table 1:** Descriptive analysis of log of indicators and their quarterly growth rates for provinces with and without the single contract

	Non single contract provinces			Single contract provinces			Difference in QGR^b^
	2017	2021	QGR^a^	2017	2021	QGR^a^	
ANC (ln)	9.66 ± 0.66	9.98 ± 0.61	2.1%***	9.51 ± 0.60	10.06 ± 0.47	3.8%***	1.7%***
Immunization (ln)	9.89 ± 0.57	10.20 ± 0.61	2.4%***	9.93 ± 0.56	10.17 ± 0.53	2.1%***	−0.3%
Nutrition consultation (ln)	10.89 ± 0.65	11.88 ± 0.70	6.7%***	10.73 ± 0.98	11.63 ± 0.84	5.9%***	−0.8%***
SBA (ln)	10.02 ± 0.65	10.29 ± 0.63	1.8%***	10.06 ± 0.51	10.41 ± 0.44	2.5%***	0.7%**
Curative visit (ln)	12.77 ± 0.54	13.02 ± 0.53	1.7%**	12.74 ± 0.56	13.13 ± 0.47	2.9%***	1.2%**
Service index (ln)	-0.41 ± 0.57	0.02 ± 0.58	2.9%***	-0.46 ± 0.62	0.03 ± 0.52	3.4%***	0.5%**
Mortality at hospitals (ln)	0.58 ± 0.29	0.45 ± 0.38	−0.7%**	0.39 ± 0.35	0.15 ± 0.34	−1.6%***	−1.0%**

The difference of the value in log form could be interpreted as percentage change. For example, in the non-single contract provinces, ln(ANC) increased from 9.66 in 2017 to 9.98 in 2021. The difference of 0.32 (9.98–9.66) means a 32% increase in volume of the ANC visits over 2017–2021.

*ANC* denotes antenatal care, *SBA* denotes skilled birth attendance, *QGR* denotes quarterly growth rate.

^a^QGR was estimated using quarterly data and random-effects model.

^b^Difference in QGR was compared to those without single contract using quarterly data and random-effects model.

*p < 0.05; **p < 0.01; ***p < 0.001.

The right side of Table 1 shows the corresponding results in the provinces with the single contract. It exhibits a similar pattern to the provinces without the single contract in that the services provided increased over time, with the largest increase in nutrition consultations of 5.9% per quarter. The smallest increase was in immunization, with a 2.1% increase per quarter. The reduction of mortality at hospitals took place at a rate of 1.6% per quarter. When compared to the QGR in the provinces without a single contract, single-contract provinces had a higher growth rate in antenatal care (ANC) (1.7%), skilled birth attendance (SBA) (0.7%), and curative visits (1.2%), but lower growth rates in immunization (0.3% lower) and nutrition consultations (0.8% lower). Mortality in the hospital was reduced faster in the single contract provinces by 0.1% per quarter. All coefficients except immunization were statistically significant (*p* < 0.05). Additionally, the difference in the baseline for all seven indicators in Table 1 was not statistically significant (*p* > 0.05). The detailed yearly data and figures showing the growth rate between the single contract and control provinces for all the indicators are presented in Supplementary Tables 1–2 and Supplementary Figs. 1–7.

### Impact of the single contract regardless of the PBF program

Table 2 shows the regression results (random-effect model) with only double interactions, demonstrating the average impact of the single contract on service delivery and mortality regardless of the implementation of the PBF program. The result indicates that the single contract was not associated with any health service delivery indicators included in the analysis because the coefficients for SC*Quarters were not statistically significant (p > 0.05). In general, the PBF program (coefficients for PBF*Quarter) was positively associated with SBA with statistical significance (*p* < 0.05). The results from fix-effect models are presented in Supplementary Table 3.

**Table 2:** The regression results of five services and mortality without the triple interaction

	ANC	Immunization	Nutrition consultations	SBA	Curative visits	Service index	Mortality
SC	0.037	0.113*	0.234***	0.075*	0.091*	0.121***	−0.021
Quarter	0.021***	0.023***	0.066***	0.018***	0.017***	0.025***	−0.007*
SC*Quarter	0.006	-0.010	−0.016	−0.0005	−0.001	−0.005	0.0001
PBF	−0.081	−0.021	0.048	0.066*	0.036	0.003	−0.004
PBF*Quarter	0.012	0.005	-0.005	0.005*	0.009	0.006	−0.010
Season2	0.013	−0.053***	−0.0039**	0.015	−0.009	−0.017	−0.023
Season3	0.028*	−0.039*	−0.012	0.034*	−0.014	−0.006	−0.066
Season4	0.023***	−0.004	0.022*	0.029***	−0.031*	0.003	−0.031
Constant	9.576***	9.920***	10.724***	9.976***	12.738***	−0.428***	0.538***

*ANC* denotes antenatal care, *SBA* denotes skilled birth attendance, *SC* denotes single contract, *PBF* denotes performance-based financing.

*p < 0.05; **p < 0.01; ***p < 0.001.

### Impact of the single contract considering the PBF program

Table 3 shows the regression results (random-effect model) after adjusting for the seasonal variation and controlling for the PBF program for the five health service indicators and the service index, as well as mortality in hospitals. We found that, in the provinces without the PBF program, the single contract did not exert any impact on service delivery, except for nutrition consultations (*p* < 0.05 for the coefficient of SC*Quarter). Nor did it exert any impact on mortality in hospitals. In the provinces with the PBF program, the single contract was associated with reduced mortality in hospitals (*p* < 0.05 for the coefficient of SC*PBF*Quarter); however, it was found to be associated with reduced immunization (*p* < 0.05 for the coefficient of SC*PBF*Quarter). The single contract exerted a positive impact on ANC, nutrition consultations, SBA, curative visits, and overall service index in PBF provinces, though not statistically significant (*P* > 0.05 for the coefficient of SC*PBF*Quarter). The results from fix-effect models are presented in Supplementary Table 4.

**Table 3:** The regression results of five services and mortality with the triple interaction

	ANC	Immunization	Nutrition consultations	SBA	Curative visits	Service index	Mortality
SC	0.031	0.122**	0.229***	0.072*	0.087*	0.119***	−0.010
Quarter	0.022***	0.022***	0.067***	0.018***	0.017***	0.026***	−0.008*
SC*Quarter	0.003	−0.005	−0.019*	−0.002	−0.003	−0.006	0.007
PBF	−0.073**	−0.032	0.054	0.069**	0.042	0.004	−0.019
PBF*Quarter	0.004	0.016**	−0.012	0.002	0.003	0.004	0.004
SC*PBF*Quarter	0.011	−0.017*	0.010	0.004	0.008	0.003	−0.021***
Season2	0.011	−0.050**	−0.042***	0.014	−0.011	−0.017	−0.019
Season3	0.026*	−0.036*	−0.014	0.033*	−0.015	−0.007	−0.063
Season4	0.022***	−0.003	0.021*	0.029***	−0.032*	-0.003	−0.029
Constant	9.579***	9.916***	10.728***	9.977***	12.740***	−0.427***	0.533***

*ANC* denotes antenatal care, *SBA* denotes skilled birth attendance, *SC* denotes single contract, *PBF* denotes performance-based financing.

*p < 0.05; **p < 0.01; ***p < 0.001.

## Discussion

Given the importance of donor funding in supporting PHC service delivery in DRC, it is critical for the health authorities, such as PHDs, to develop mechanisms to align funding from development partners to the local population’s health priorities. The results show that the single contract alone had no direct impact on PHC services, which may be mediated by other health system factors.

The no impact of the single contract alone on service delivery may have to do with the challenges faced during the single contract implementation and the fact that it is restricted to support certain pillars of the health system (e.g., governance), instead of holistic key foundations of the health system faced by the country, such as shortage of funding, human resources for health, and inadequate health infrastructure^12–15^. The effect of the single contract on service delivery is likely to be non-linear, as a policy implementation is often mediated by a range of health system factors, including government capacity, absorptive ability, supervision quality, and fiscal space^16,17^. For provinces that have a budget that is unrelated to the Operational Action Plan (OAP) or the National Health Development Plan, the single contract has instilled the concept of traceability of resources in its implementation. Theoretically, provinces with the single contract, based on the agreement, need to monitor the funding and activities carried out under the province’s OAP every three months and provide prompt feedback to health facilities to improve health services. However, the PHDs had a poor budget execution rate for both donor and government budgets^18,19^, which hindered the operation of the single contract. The weak public financial management capacity contributes to the doubts that development partners have in the ability of provinces to properly manage their funding^4,20^. Thus, it affects the acceptance among development partners of this approach to promote funding alignment.

The lack of monitoring and evaluation (M&E) mechanisms could be another implementation issue of the single contract. The single contact requires PHDs to conduct regular visits to health facilities to identify the bottlenecks that prevent populations from effective access to health care– particularly essential health services– and accordingly develop action plans based on the visit. It is also suggested that the single contract would allow for better coaching of the provinces and better implementation of associated activities at PHDs for service delivery. However, many provinces with a single contract have mediocre activity/performance and coaching rates^11^. More specifically, the activity completion or performance rate between 2018 and 2019 was below 55% in the provinces of Maniema, Mongala, South Kivu, and Tshuapa. The coaching rate also remained low in Mai Ndome, Mongala, and Tshuapa (based on calculations by the authors). The low-performance rate in these provinces has not been used for correcting PHD’s performance concerns. Future reforms of the single contract program should hold PHDs more accountable for the performance of implementation activities.

Despite the single contract’s potential to enhance PHDs’ transparency, accountability, and capacity to coordinate and plan health activities, several implementation areas should be strengthened, including technical capacity at the PHD, M&E, and development partners’ involvement^21,22^. Specifically, to improve the leadership and technical capacity at the PHD, the central government and development partnership could help identify capacity gaps and provide tailored technical assistance to the officials at the PHD, particularly on project coordination, budgeting and planning, service supervision, and leadership. To enhance M&E, besides the regular monitoring duties and obligations specified in the contract, it is important to make use of existing quarterly monitoring data collected. The planning unit at the central level needs to systematically enter programmatic and financial data in the health management information system to monitor contract implementation and fund absorption, analyze them and disseminate the results among key stakeholders. Further, holding development partners accountable would help better engage them in participating in provincial health planning and coordination^7^.

Surprisingly, the implementation of the single contract is associated with a lower growth rate of nutrition consultation. DRC experiences severe malnutrition problems, with a stunting rate of 42% among children under 5 in 2018^23^. To address the nutrition concern, development partners have mobilized substantial resources. In 2019, the World Bank approved a multisectoral nutrition and health project of US$502 million in DRC^24^. Aside from the World Bank, many other actors are involved in food security, nutrition financing, and service delivery. The project implementation was not necessarily equally balanced between provinces with and without the single contract. For example, between 2019 and 2020, a program focused on providing treatments to children under 5 years old with severe wasting was only implemented in Kasai, a province without the single contract^25^. The existence of such programs in non-single contract provinces may bias the results unfavorably for the single contract. Additionally, two provinces are supported by humanitarian aid in the single contract arm: North Kivu and South Kivu. It was reported that the nutrition status (e.g., stunting) in these two areas has improved^26^. The improved nutrition status among the population in the single contract provinces is likely to necessitate fewer nutrition consultation visits to health facilities. This may contribute to the lower growth rate of nutrition consultation visits in the single contract provinces. To improve nutrition services, donors should continue collaborating with PHDs and engage community stakeholders. Some actions include raising the awareness of nutrition concerns among children, monitoring children growth through outreach programs, and ensuring that treatment resources and facility are available in accessible health facilities^27,28^.

It is important to note that the single contract has a favorable synergy with the PBF program on delivering PHC services. In 2016, with financial support from the World Bank, DRC launched *projet d’Appui des Services de Sante* (PDSS). Similar to PBF programs implemented by the World Bank in other countries, the PBF program provides financial incentives to health providers (health clinics and hospitals) based on a set of pre-determined health indicators, including PHC indicators^29^. The PBF program has shown a favorable impact on PHC services in DRC and other countries^14,30,31^. The PBF program is often treated as a health system intervention to strengthen accountability (e.g., reporting, monitoring, and evaluation) of health providers. The favorable synergy between PBF and the single contract enhances the need to address potential implementation barriers (e.g., M&E and human resources) of the single contract. The supervision function of PHDs through the single contract would work better when health facilities are mobilized.

Several limitations of this study should be acknowledged. Firstly, this study does not include a pure control group that has similar characteristics as those provinces with the single contract. The provinces in the control group were assigned by the government and therefore did not perfectly match with those in the intervention group. Generally, the potential bias from the non-randomization assignment often poses a great concern in cross-sectional studies. In a study with panel datasets, the nature of panel data analysis, particularly the fixed-effect model that focuses on the within-group variation, could significantly mitigate the concern resulting from the non-randomization^32^. Secondly, the implementation of the single contract program was not consistent. The single contract was renewed on an annual basis. Some provinces stopped the implementation of the single contract without/with the resumption of the implementation. Although we tracked the implementation status for each quarter, we could not quantify the tail impact of the implementation when the single contract stopped. Thirdly, it is likely that other factors than the single contract and PBF program could affect health service delivery, and the statistical model may run into omitted variable bias. However, the use of the panel data to examine the impact again mitigates such a concern, as time-invariant unobserved provincial characteristics were controlled in the model automatically. Fourthly, our data were aggregated at the provincial level, and it overlooked within-province heterogeneity. Lastly, the single contract is likely to influence processes of service delivery, such as transparency, coordination, accountability, and supervision, which fall beyond direct service measures. The impact of the single contract should be interpreted within the broad implementation context. Identifying and addressing implementation barriers and building a favorable policy implementation ecosystem would help maximize the single contract’s impact. Despite these limitations, this study provides valuable information on where to potentially improve the single contract mechanism for a better PHC system in DRC.

This study shows that the single contract alone had a no impact on the primary healthcare service delivery. When combined with the PBF program, the single contract contributed to a reduced hospital mortality rate, while also positively affecting other PHC indicators except immunization. This study highlights the importance of understanding and effectively addressing the single contract’s implementation barriers. In summary, the single contract, as an important governance instrument, create an enabling environment to strengthen supervision and resource coordination of PHDs. However, it effects on service delivery hinge on broader health ecosystem, such as effective M&E, strong absorptive and implementation capacity, robust public finance management, adequate fiscal space, and well-aligned incentives.

## Methods

### Research design

To quantitatively assess the impact of the single contract on health service delivery, we used a quasi-experimental research design to evaluate the single contract’s effect on the key maternal and child health services^33^, where the unit of the analysis was at the provincial level and the intervention under examination was whether a province implemented the single contract in a particular quarter or not.

**Fig. 2:**
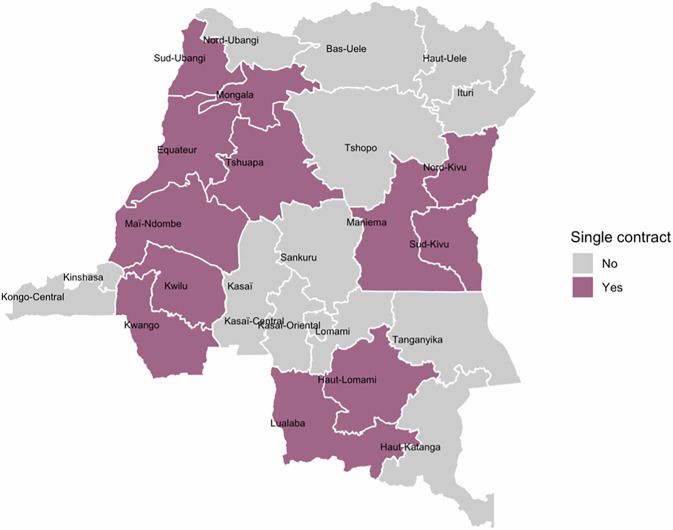
The provinces implementing the single contract and their controls.

As a part of a 5 year World Bank-funded project --- *projet d’Appui des Services de Sante (PDSS)*--- implemented in DRC from 2017 to 2022, the single contract was planned to start in 2017 in 12 provinces out of the total 26 provinces in the country. However, the assignment of the single contract was not random and was based on a joint decision by the Ministry of Health and the World Bank. The actual implementation of the single contract did not start until 2018 and was not continuously implemented over time. For example, Sud-Kivu started and implemented the single contract in 2018, but stopped the program in 2019. The provinces that implemented the single contract program for at least one year are shown in Fig. 2. For details implementation status for each year, please see Supplementary Table 5.

### Measurements

The indicators used for the impact evaluation were from the World Bank-funded project that reported service delivery data on PHC services for all 26 provinces^33^. The available evaluation indicators were: (1) the number of pregnant women having at least four ANC visits before delivery; (2) the number of women and children who have received basic nutrition consultations; (3) the number of children aged between 12 and 24 months fully immunized; (4) the number of deliveries attended by SBA; (5) the number of curative visits; and (6) the 48-hour in-hospital mortality rate (quarterly) in the Department of Obstetrics and Gynecology (OB-GYN). These indicators were routinely reported by all the health facilities in the country through its information system monthly. The associated data, including the implementation status of the single contract, were then aggregated at the provincial level. To ensure data stability, we aggregated the data on a quarterly basis. The final dataset contained the quarterly data for the above six indicators from January 2017 through June 2021 (18 quarters) for all 26 provinces, with a total of 468 quarter-provinces.

Besides examining the individual indicators, we generated an indicator of the service index, the geometric mean of the relative value to the average of the first five services measured above, to measure the overall level of service delivery. This indicator excluded the mortality rate at the OB-GYN department as it was not a service delivery indicator. Ethical approval is not applicable because the study used facility-level data to perform the quantitative analysis.

### Statistical analysis

The data were first verified by local researchers in DRC for accuracy. Researchers at Washington DC conducted a descriptive analysis of the data to identify outliers (3 times the standard deviation from the mean) and reshared them with the local team for verification.

We conducted descriptive analyses of core outcome indicators and calculated the mean and standard deviation for the services and the service index by quarter for single contract and non single contract provinces. Given that the data were aggregated by quarter, we calculated the QGR for each of services, the service index, and mortality at hospitals for the two arms by fitting linear lines between the logarithmic form of the heath output indicators against quarters. We then calculated the difference in QGR between the two arms. The logarithmic transformation facilitated the estimation and comparison of QGR betwteen the provinces with the single contract and those without.

With the clear time trend of the health output indicators and the large number of panels (18 quarters), we quantified the impact of the single contract on service delivery by comparing the QGR of the service delivery between the period with the single contract and that without in this study. Thus, we used the following statistical model to control for seasonal variations and individual province impact, as well as the PBF program that was implemented in selected provinces in DRC:

1 $$\begin{array}{lll}{\mathrm{log}}\left(service{s}_{it}\right)&=&\,{\beta }_{0}+\,{\beta }_{1}{\mathrm{SC}}_{it}+\,{\beta }_{2}\,Quarter+\,{\beta }_{3}{\mathrm{SC}}_{it}* quarter\\ &&+{\beta }_{4}PB{F}_{it}+\,{\beta }_{5}PB{F}_{it}* quarter+{\beta }_{6}{\mathrm{SC}}_{it}* PB{F}_{it}* quarter\\ &&+\,{\beta }_{7}\,{season2}_{it}+{\beta }_{8}{season3}_{it}+\,{\beta }_{9}{season4}_{it}+\,{\alpha }_{i}+\,{\varepsilon }_{it}\end{array}\,\,$$

where services_it_ represents the six services and the service index; SC_*it*_ shows where the data is from– i.e. the provinces where the single contract was implemented (SC = 1) or not (SC = 0) for province *i* at quarter *t*; *quarter* is a continuous variable with Jan 2017 as 1; SC*quarter is the interaction term between the *single contract* and *quarter*; *PBF* and PBF*quarter represent whether the provinces implemented the PBF program (PBF = 1) or not (PBF = 0) and its interaction with quarter; SC*PBF*quarter shows the interaction of single contract, PBF, and quarters; season2, season3, and season4 indicate whether the data were for the second, third or fourth season; ${\alpha }_{i}$ represents province individual impact; and ${\varepsilon }_{{it}}$ represents the random noise of the model. To correct potential serial correlation among observations within a province, the model clustered the standard errors at the province level. Our data contained 18 quarters, and the panel data approach controlled for some unobserved variables. Because we treated the time variable – quarters – as a continuous variables, no time fixed/random time effect was included in the model. This is similar to the model used in studies to examine the policy impact in Haiti and Afghansitan^30,34^.

The coefficient of ${\beta }_{3}$ shows the differential growth rate per quarter between the single contract and control group without PBF. The impact of the single contract with PBF is the sum of ${\beta }_{3}$ and ${\beta }_{6}$. The impact of PBF without the single contract is captured by ${\beta }_{5},$ and the impact of PBF with the single contract is the sum of ${\beta }_{5}$ and ${\beta }_{6}$. A positive value of βs suggests a higher growth rate of the service under examination.

To examine the overall impact of the single contract, we used the random-effect model without the triple interaction of SC*PBF*quarters. In this case, the coefficient of ${\beta }_{3}$ captures the average effect of the single contract in the sample. Similarly, the coefficient of ${\beta }_{5}$ captures the average effect of the PBF. For all the analyses, we did not adjust for the population size of provinces as we examined more within-province variation using panel data analysis approaches.

Please note that the model controlled for the PBF program. The PBF program provided health providers (health facilities) in selected provinces with financial incentives to reward health service delivery while health facilities in the control provinces receive a lump sum of quarterly payments that were not tied to health facilities’ performance. The details of the program could be found elsewhere^14^. The status of PBF implementation at the provincial level was recorded every quarter and included in the analysis as a control variable. Given that the PBF and single contract were implemented intermittently in many provinces, the implementation of PBF and the single contract did not perfectly overlap. This provided a great opportunity to examine their independent impact and interaction on the PHC service delivery. As previously mentioned, we obtained the implementation status of PBF and the single contract by quarter.

We conducted the statistical analysis using both the fixed-effect and random-effect models and compared the results using the Hausman test. The differences between the two models were not statistically significant. Thus, we report the findings from the random-effects model. To examine the overall impact of the single contract, we used the random-effects model without the triple interaction of SC*PBF*quarters.

We used 2017 data to test the parallel trends assumption and found that the growth rates in the single-contract and non-single-contract arms were comparable before the policy’s implementation (Supplementary Table 6). Additionally, we performed a robustness test of the impact assessment regression models using the population sizes of the provinces as weights (Supplementary Tables 7−8). All the statistical analyses were conducted using R 4.3.0 (R Foundation, Vienna, Austria)

## Supplementary information

Supplementary Information

## Data Availability

All the data used for the analysis and the coding to perform the analysis are available upon request.
