# Supplementary material for: The impact of the single contract on health service delivery in the Democratic Republic of the Congo (DRC): findings from a quasi-experimental study
Source: Npj Health Syst. 2025 Dec 16;2:50. doi: 10.1038/s44401-025-00053-0 (PMC13354146; doi:10.1038/s44401-025-00053-0)
Supplement: Supplementary file 1 — Supplementary Information [file 44401_2025_53_MOESM1_ESM.docx]

Supplementary information

Supplementary Tables

Supplementary Table 1. Descriptive analysis of log of indicators and their quarterly growth rates for provinces without the single contract

|  | Non single contract provinces | | | | | |
| --- | --- | --- | --- | --- | --- | --- |
|  | 2017 | 2018 | 2019 | 2020 | 2021 | QGR |
| ANC (ln) | 9.66±0.66 | 9.77±0.64 | 9.83±0.62 | 9.94±0.60 | 9.98±0.61 | 2.1%*** |
| Immunization (ln) | 9.89±0.57 | 10.04±0.56 | 10.14±0.53 | 10.22±0.54 | 10.20±0.61 | 2.4%*** |
| Nutrition visit (ln) | 10.89±0.65 | 11.22±0.59 | 11.45±0.54 | 11.71±0.65 | 11.88±0.70 | 6.7%*** |
| SBA (ln) | 10.02±0.65 | 10.12±0.64 | 10.19±0.61 | 10.26±0.60 | 10.29±0.63 | 1.8%*** |
| Curative visit (ln) | 12.77±0.54 | 12.86±0.56 | 12.94±0.53 | 12.99±0.51 | 13.02±0.53 | 1.7%** |
| Service index (ln) | -0.41±0.57 | -0.26±0.56 | -0.15±0.53 | -0.03±0.55 | 0.02±0.58 | 2.9%*** |
| Mortality at hospitals (ln) | 0.58±0.29 | 0.53±0.36 | 0.53±0.32 | 0.49±0.37 | 0.45±0.38 | -0.7%** |

Note: ANC denotes antenatal care; SBA denotes skilled birth attendance; *p< 0.05; **p<0.01; ***p<0.001.

Supplementary Table 2.Descriptive analysis of log of indicators and their quarterly growth rates for provinces with the single contract

|  | Single contract provinces | | | | | | |
| --- | --- | --- | --- | --- | --- | --- | --- |
|  | 2017 | 2018 | 2019 | 2020 | 2021 | QGR |  |
| ANC (ln) | 9.51±0.60 | 9.71±0.51 | 9.87±0.48 | 10.02±0.47 | 10.06±0.47 | 3.8%*** |  |
| Immunization (ln) | 9.93±0.56 | 10.05±0.58 | 10.17±0.50 | 10.23±0.5 | 10.17±0.53 | 2.1%*** |  |
| Nutrition visit (ln) | 10.73±0.98 | 11.13±0.86 | 11.31±0.87 | 11.48±0.85 | 11.63±0.84 | 5.9%*** |  |
| SBA (ln) | 10.06±0.51 | 10.22±0.45 | 10.31±0.46 | 10.41±0.44 | 10.41±0.44 | 2.5%*** |  |
| Curative visit (ln) | 12.74±0.56 | 12.93±0.51 | 13.03±0.54 | 13.15±0.49 | 13.13±0.47 | 2.9%*** |  |
| Service index (ln) | -0.46±0.62 | -0.25±0.56 | -0.12±0.55 | 0.00±0.53 | 0.03±0.52 | 3.4%*** |  |
| Mortality at hospitals (ln) | 0.39±0.35 | 0.30±0.40 | 0.28±0.36 | 0.16±0.29 | 0.15±0.34 | -1.6%*** |  |

Note: ANC denotes antenatal care; SBA denotes skilled birth attendance; QGR denotes quarterly growth rate; *p< 0.05; **p<0.01; ***p<0.001.

Supplementary Table 3. The regression results of five services and mortality without the triple interaction (Fixed-effects model)

| Variable | ANC | Immunization | Nutrition | SBA | Curative | Serv index | Mortality |
| --- | --- | --- | --- | --- | --- | --- | --- |
| SC | 0.037 | 0.113* | 0.233** | 0.074 | 0.091 | 0.120** | -0.013 |
| Quarter | 0.021*** | 0.023*** | 0.066*** | 0.018*** | 0.017*** | 0.025*** | -0.007* |
| SC*Quarter | 0.006 | -0.010 | -0.016 | 0.0005 | -0.001 | -0.005 | 0.001 |
| PBF | -0.078 | -0.018 | 0.057 | 0.067* | 0.04 | 0.006 | -0.016 |
| PBF*Quarter | 0.012 | 0.005 | -0.005 | 0.005 | 0.009 | 0.006 | -0.011 |
| Season2 | 0.013 | -0.053** | -0.039** | 0.015 | -0.009 | -0.017 | -0.024 |
| Season3 | 0.028* | -0.039* | -0.012 | 0.034* | -0.014 | -0.006 | -0.067 |
| Season4 | 0.023*** | -0.004 | 0.022* | 0.029*** | -0.031 | -0.003 | -0.032 |
| Constant | 9.575*** | 9.919*** | 10.720*** | 9.976*** | 12.736*** | -0.429*** | 0.543*** |

Note: ANC denotes antenatal care; SBA denotes skilled birth attendance; SC denotes single contract; PBF denotes performance-based financing; *p< 0.05; **p<0.01; ***p<0.001.

Supplementary Table 4. The regression results of five services and mortality with a triple interaction (Fixed-effects model)

| Variable | ANC | Immunization | Nutrition | SBA | Curative | Serv index | Mortality |
| --- | --- | --- | --- | --- | --- | --- | --- |
| SC | 0.031 | 0.121* | 0.227** | 0.072 | 0.086* | 0.119** | -0.004 |
| Quarter | 0.022*** | 0.022*** | 0.067*** | 0.018*** | 0.017*** | 0.026*** | -0.008* |
| SC*Quarter | 0.003 | -0.005 | -0.019* | -0.002 | -0.004 | -0.006 | 0.007 |
| PBF | -0.07 | -0.028 | 0.064 | 0.070* | 0.046 | 0.008 | -0.028 |
| PBF*Quarter | 0.004 | 0.015* | -0.013 | 0.002 | 0.002 | 0.004 | 0.001 |
| SC*PBF*Quarter | 0.011 | -0.016 | 0.011 | 0.004 | 0.009 | 0.003 | -0.018** |
| Season2 | 0.011 | -0.050** | -0.041** | 0.014 | -0.011 | -0.017 | -0.021 |
| Season3 | 0.027* | -0.036* | -0.013 | 0.033* | -0.015 | -0.007 | -0.064 |
| Season4 | 0.022*** | -0.003 | 0.021* | 0.029*** | -0.032* | -0.003 | -0.03 |
| Constant | 9.578*** | 9.915*** | 10.723*** | 9.977*** | 12.738*** | -0.428*** | 0.538*** |

Note: ANC denotes antenatal care; SBA denotes skilled birth attendance; SC denotes single contract; PBF denotes performance-based financing; *p< 0.05; **p<0.01; ***p<0.001.

Supplementary Table 5. Single contract categorization and its implementation status from 2017-2021

| Provinces | Single contract group (0 = no, 1 = yes) | Single contract implementation status (0 = no, 1 = yes) | | | | |
| --- | --- | --- | --- | --- | --- | --- |
|  |  | 2017 | 2018 | 2019 | 2020 | 2021 |
| Bas-Uele | 0 | 0 | 0 | 0 | 0 | 0 |
| Equateur | 1 | 0 | 1 | 0 | 0 | 0 |
| Haut-Katanga | 1 | 0 | 0 | 0 | 1 | 1 |
| Haut-Lomami | 1 | 0 | 1 | 1 | 1 | 1 |
| Haut-Uele | 0 | 0 | 0 | 0 | 0 | 0 |
| Ituri | 0 | 0 | 0 | 0 | 0 | 0 |
| Kasaï | 0 | 0 | 0 | 0 | 0 | 0 |
| Kasaï-Central | 0 | 0 | 0 | 0 | 0 | 0 |
| Kasaï-Oriental | 0 | 0 | 0 | 0 | 0 | 0 |
| Kinshasa | 0 | 0 | 0 | 0 | 0 | 0 |
| Kongo-Central | 0 | 0 | 0 | 0 | 0 | 0 |
| Kwango | 1 | 0 | 1 | 1 | 1 | 1 |
| Kwilu | 1 | 0 | 1 | 1 | 1 | 1 |
| Lomami | 0 | 0 | 0 | 0 | 0 | 0 |
| Lualaba | 1 | 0 | 1 | 1 | 1 | 1 |
| Maï-Ndombe | 1 | 0 | 1 | 1 | 1 | 1 |
| Maniema | 1 | 0 | 0 | 1 | 1 | 1 |
| Mongala | 1 | 0 | 1 | 1 | 1 | 1 |
| Nord-Kivu | 1 | 0 | 1 | 1 | 1 | 1 |
| Nord-Ubangi | 0 | 0 | 0 | 0 | 0 | 0 |
| Sankuru | 0 | 0 | 0 | 0 | 0 | 0 |
| Sud-Kivu | 1 | 0 | 1 | 0 | 0 | 0 |
| Sud-Ubangi | 1 | 0 | 1 | 1 | 1 | 1 |
| Tanganyika | 0 | 0 | 0 | 0 | 0 | 0 |
| Tshopo | 0 | 0 | 0 | 0 | 0 | 0 |
| Tshuapa | 1 | 0 | 1 | 1 | 1 | 1 |

Supplementary Table 6. Test of parallel trends prior to 2018

|  | ANC | Immunization | Nutrition consultations | SBA | Curative visits | Service index | Mortality |
| --- | --- | --- | --- | --- | --- | --- | --- |
| Constant | 9.6357*** | 9.7500*** | 10.7101*** | 9.9867*** | 12.7183*** | -0.4968*** | 0.5564*** |
| SC | -0.192 | 0.05631 | -0.1757 | -0.00155 | -0.0831 | -0.0792 | -0.0967 |
| Quarter | 0.0147 | 0.09108 | 0.1182 | 0.02487 | 0.035 | 0.0568 | 0.016 |
| SC*Quarter | 0.0297 | -0.00843 | 0.0112 | 0.02624 | 0.036 | 0.0189 | -0.0635 |

Note: ANC denotes antenatal care; SBA denotes skilled birth attendance; SC denotes single contract; PBF denotes performance-based financing; ***p<0.001.

Supplementary Table *7*. Results from weighted random-effects models without triple interaction

| Variable | ANC | Immunization | Nutrition | SBA | Curative | Serv index | Mortality |
| --- | --- | --- | --- | --- | --- | --- | --- |
| SC | -0.006 | 0.158*** | 0.222*** | 0.044* | 0.071* | 0.103*** | 0.023 |
| Quarter | 0.019*** | 0.021*** | 0.068*** | 0.017*** | 0.016*** | 0.025*** | -0.005* |
| SC*Quarter | 0.010*** | -0.015*** | -0.017*** | 0.002 | 0.001 | -0.004. | -0.004 |
| PBF | -0.056* | -0.098* | 0.072 | 0.061* | 0.047 | -0.003 | -0.026 |
| PBF*Quarter | 0.009*** | 0.011*** | -0.01* | 0.005** | 0.007** | 0.005** | -0.008* |
| Season2 | 0.025* | -0.037* | -0.025 | 0.023* | -0.006 | -0.006 | -0.04. |
| Season3 | 0.041*** | -0.026 | 0.013 | 0.048*** | -0.015 | 0.006 | -0.072** |
| Season4 | 0.028* | 0.003 | 0.022 | 0.034** | -0.041** | -0.004 | -0.038 |
| Constant | 9.586*** | 9.931*** | 10.714*** | 9.98*** | 12.751*** | -0.42*** | 0.526*** |

Note: ANC denotes antenatal care; SBA denotes skilled birth attendance; SC denotes single contract; PBF denotes performance-based financing; *p< 0.05; **p<0.01; ***p<0.001.

Supplementary Table *8*. Results from weighted random-effects models with triple interaction

|  | ANC | Immunization | Nutrition consultation | SBA | Curative visits | Service index | Mortality |
| --- | --- | --- | --- | --- | --- | --- | --- |
| SC | -0.001 | 0.152*** | 0.226*** | 0.045* | 0.075* | 0.104*** | 0.015 |
| Quarter | 0.020*** | 0.02*** | 0.069*** | 0.017*** | 0.017*** | 0.025*** | -0.006** |
| SC*Quarter | 0.005* | -0.008* | -0.022*** | 0.001 | -0.003 | -0.006* | 0.005 |
| PBF | -0.060* | -0.093* | 0.069 | 0.06* | 0.044 | -0.004 | -0.02 |
| PBF*Quarter | 0.002 | 0.021*** | -0.018*** | 0.003 | 0.001 | 0.003 | 0.004 |
| SC*PBF*Quarter | 0.013*** | -0.018*** | 0.014* | 0.004 | 0.010** | 0.004 | -0.022*** |
| Season2 | 0.023* | -0.034* | -0.027 | 0.022* | -0.007 | -0.006 | -0.036 |
| Season3 | 0.040*** | -0.024 | 0.011 | 0.048*** | -0.017 | 0.006 | -0.07** |
| Season4 | 0.027* | 0.005 | 0.021 | 0.034** | -0.042** | -0.004 | -0.036 |
| Constant | 9.584*** | 9.934*** | 10.711*** | 9.98*** | 12.7*** | -0.421*** | 0.528*** |

Note: ANC denotes antenatal care; SBA denotes skilled birth attendance; SC denotes single contract; PBF denotes performance-based financing; *p< 0.05; **p<0.01; ***p<0.001.

Supplementary Figures


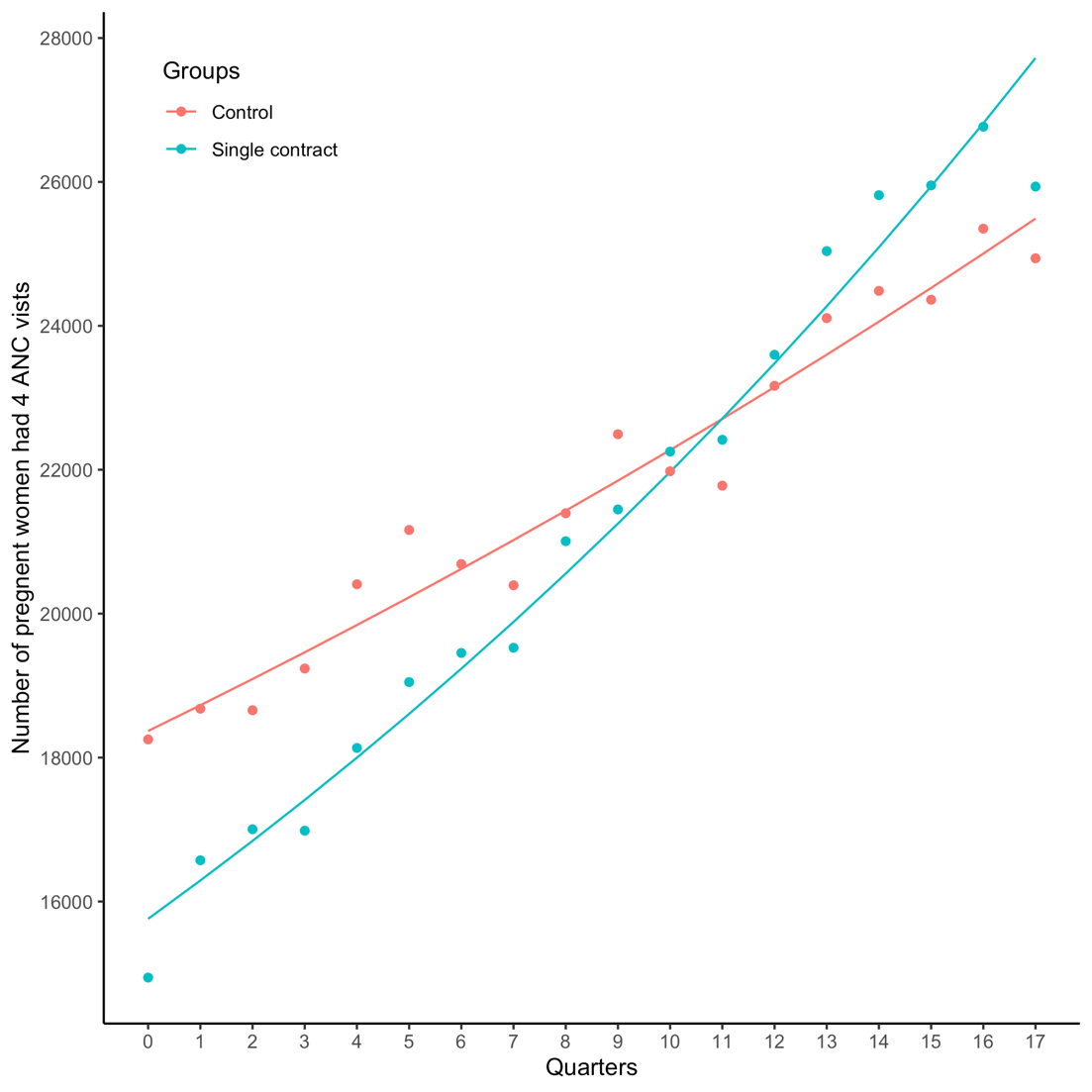


Supplementary Figure 1. ANC visits between single contract and control provinces


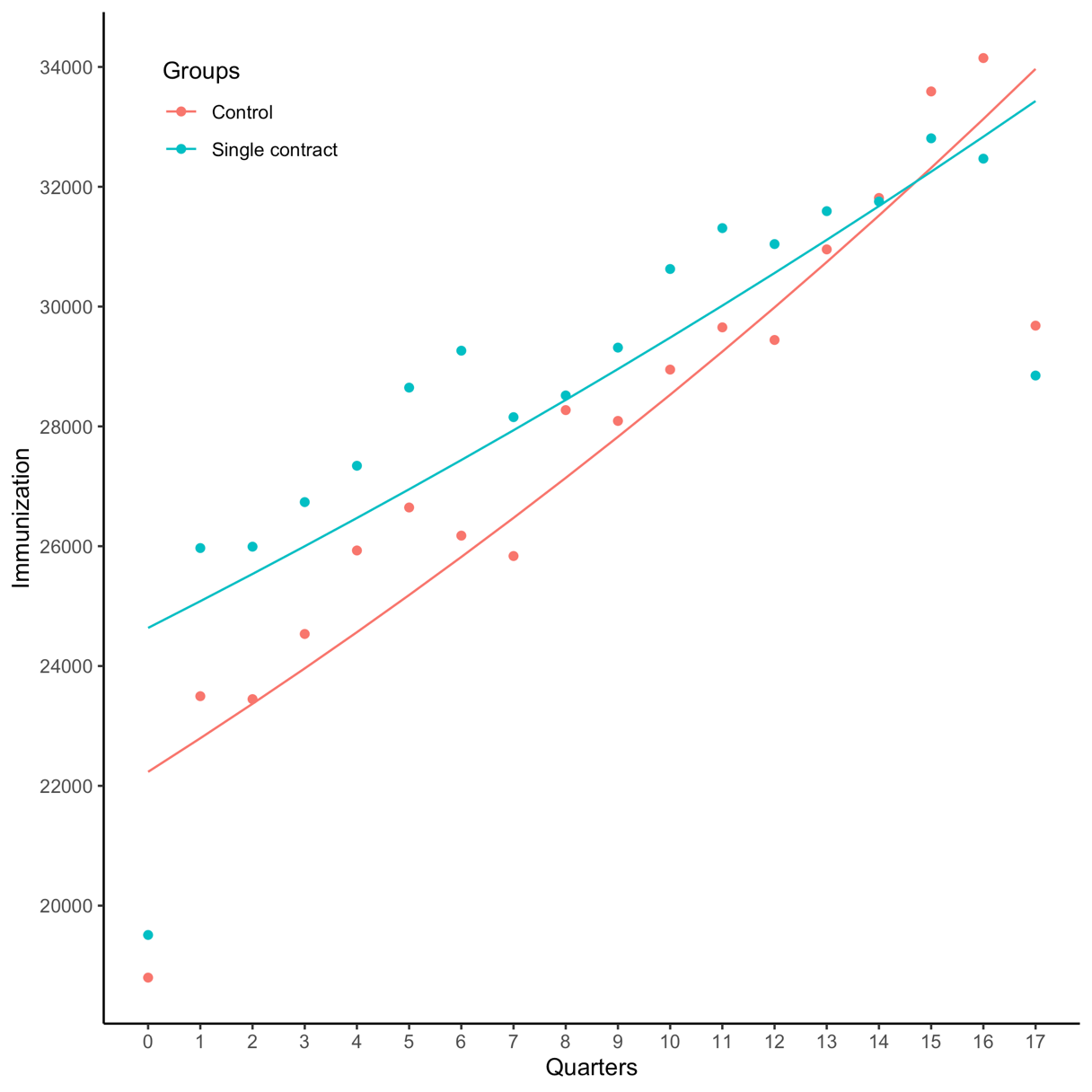


Supplementary Figure 2. Number of children immunized between single contract and control provinces


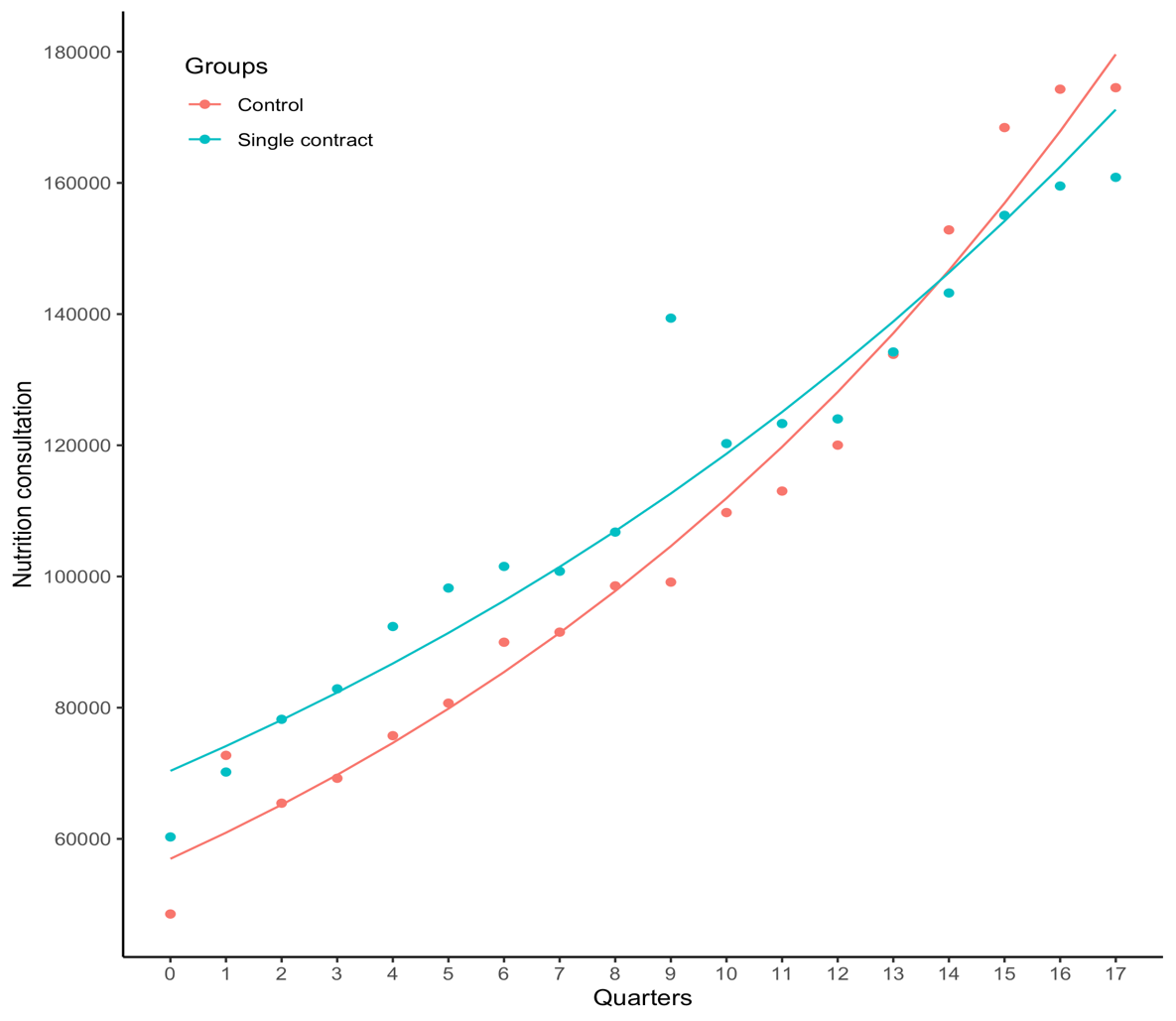


Supplementary Figure 3. Number of nutrition visits between single contract and control provinces


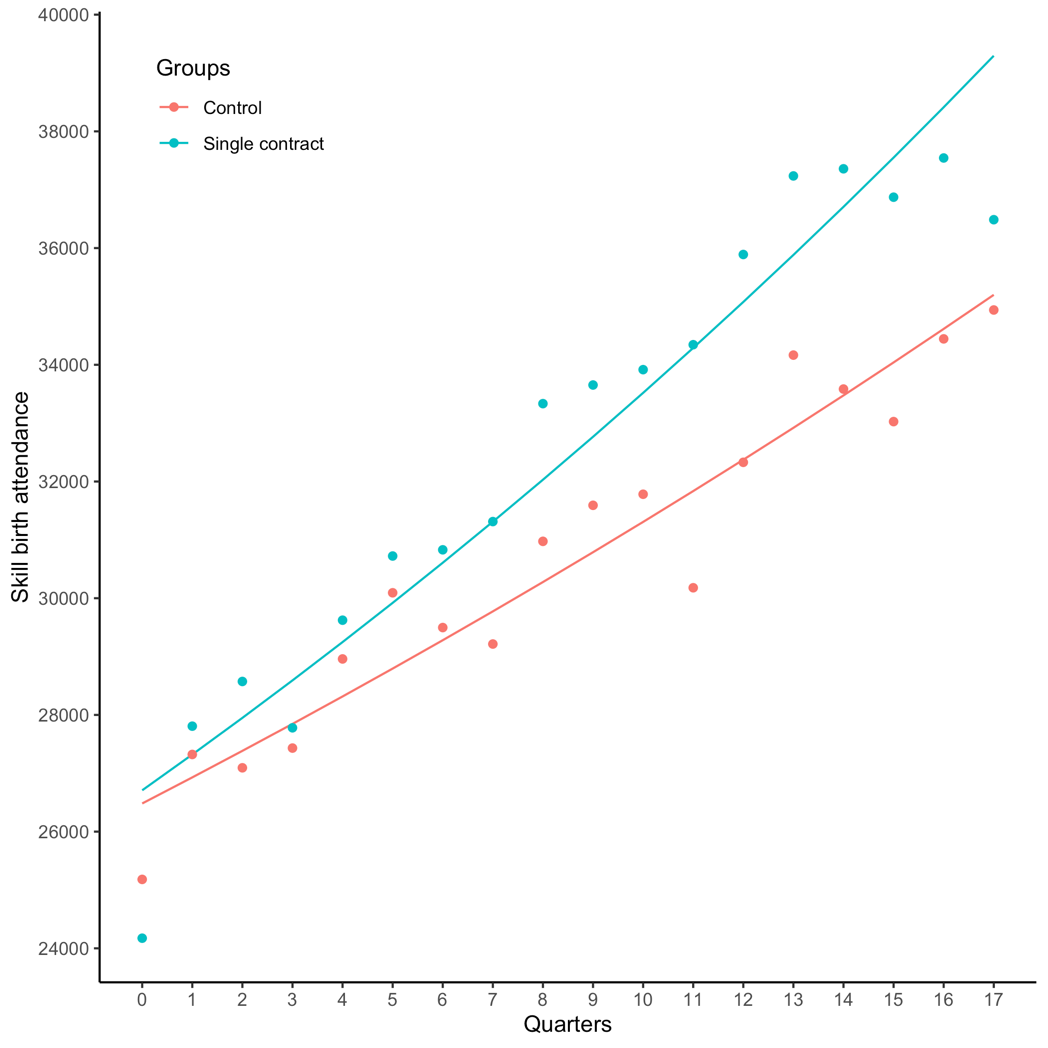


Supplementary Figure 4. Number of pregnant women giving birth attended by health professionals between single contract and control provinces


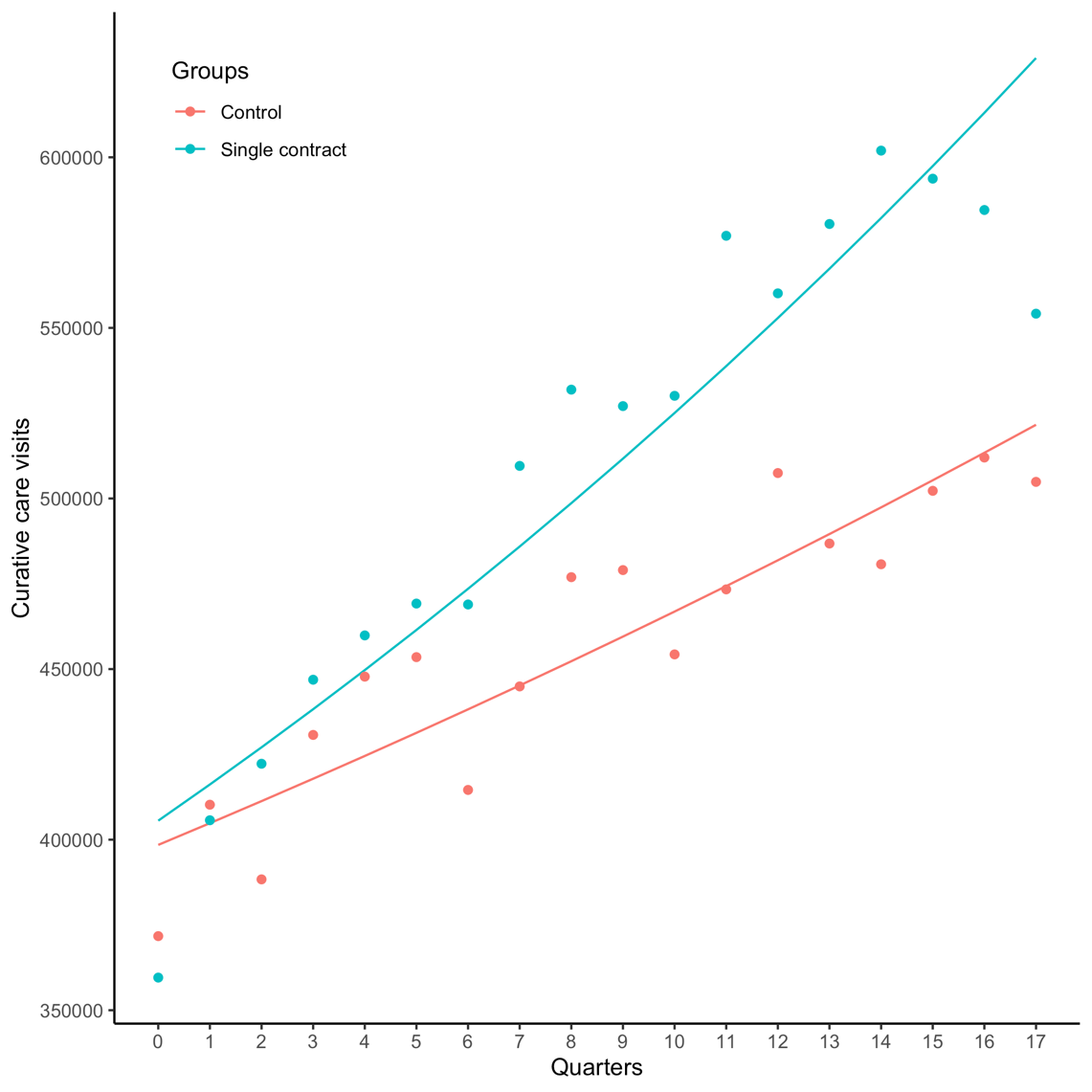


Supplementary Figure 5. Number of curative visits between single contract and control provinces


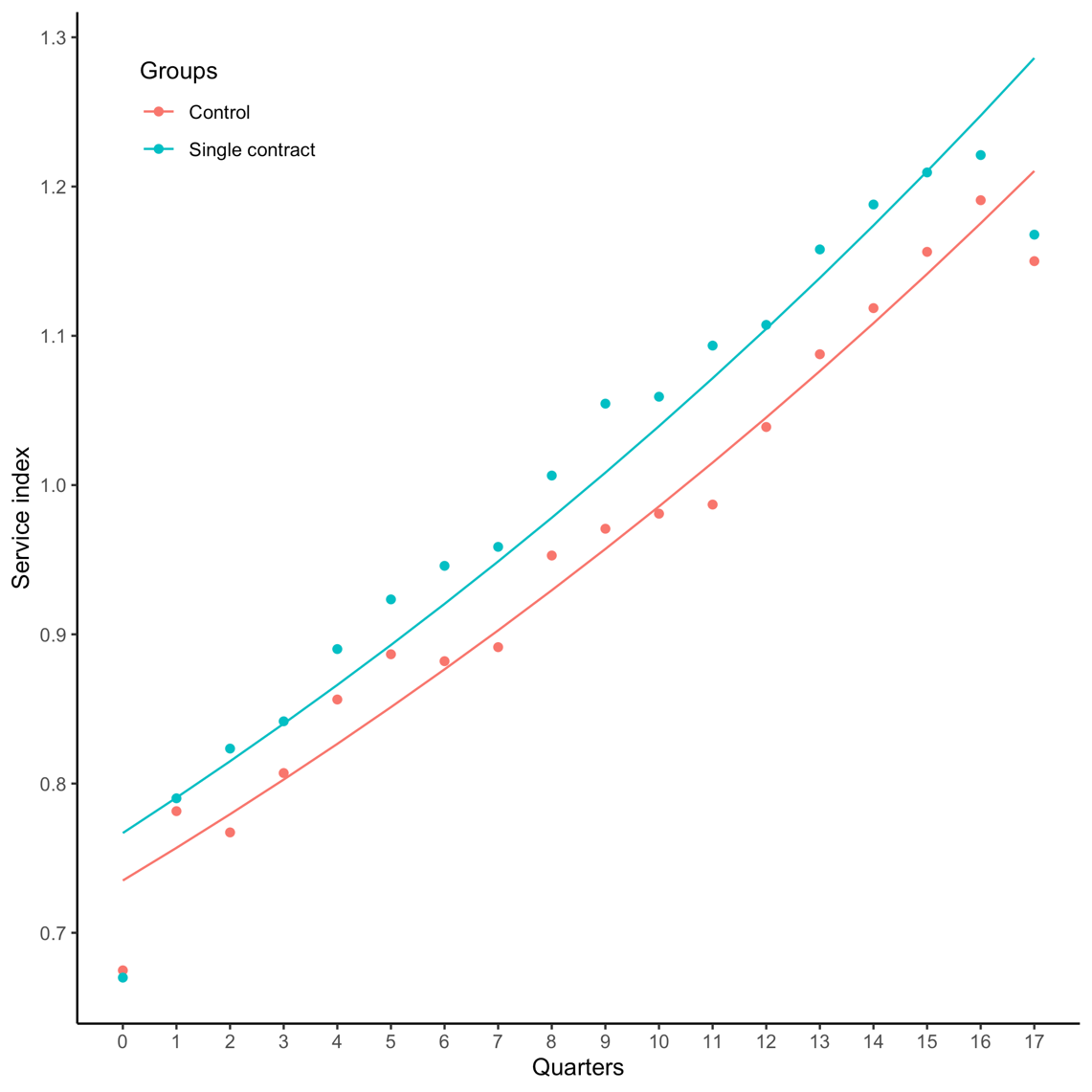


Supplementary Figure 6. Service index between single contract and control provinces


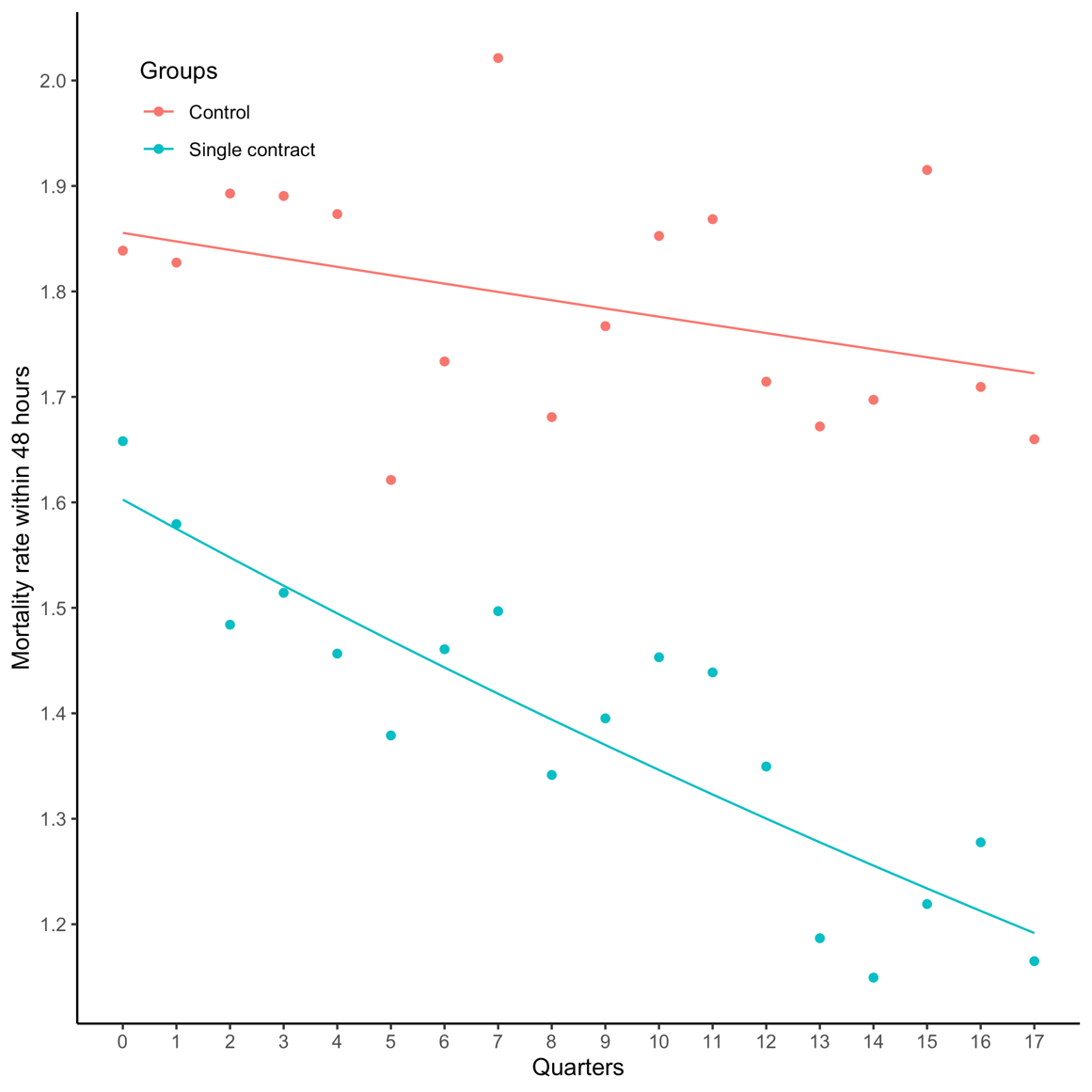


Supplementary Figure 7. The 48-hour in-hospital mortality rate in the Department of Obstetrics and Gynecology (OB-GYN) between single contract and control provinces
